# Sleep disturbance and social reward processing as characteristics linking minority victimization and suicidal ideation in youth

**DOI:** 10.3389/fnins.2024.1475097

**Published:** 2025-01-07

**Authors:** T. H. Stanley Seah, Kristen L. Eckstrand, Tina Gupta, Lily C. X. Jensen, Zachary M. Brodnick, Chloe M. Horter, Alice M. Gregory, Peter L. Franzen, Michael P. Marshal, Erika E. Forbes

**Affiliations:** ^1^Department of Psychiatry, University of Pittsburgh, Pittsburgh, PA, United States; ^2^Department of Psychology, Harvard University, Cambridge, MA, United States; ^3^Department of Psychology, Royal Holloway, University of London, Egham, United Kingdom; ^4^Department of Psychology, University of Pittsburgh, Pittsburgh, PA, United States; ^5^Department of Clinical and Translational Science, University of Pittsburgh, Pittsburgh, PA, United States; ^6^Department of Pediatrics, University of Pittsburgh, Pittsburgh, PA, United States

**Keywords:** adolescence, sleep, social stress, suicide, social reward, neuroimaging, victimization, health disparities

## Abstract

Adolescence is characterized by heightened sleep disturbances (e.g., poor sleep quality and irregular/insufficient sleep) and sensitivity to social feedback that may exacerbate suicidal ideation (SI). Victimization experiences (e.g., bullying, humiliation) can contribute to sleep disturbances and SI, particularly among minoritized youth (e.g., sexual/gender, racial/ethnic minorities). However, sensitivity to social reward, despite social challenges, may buffer against the effects of victimization on sleep and SI. In a diverse sample of youth at varying suicide risk, we examined sleep disturbance as a mediator of victimization and SI, and if neural response to social reward moderated the link between victimization and sleep disturbance. Ninety eight youth (14–22 years old; 50% sexual and/or racial/ethnic minority) with varying SI severity provided self-report data on past-six-month identity-related victimization, past-week sleep disturbance, and past-month SI. Seventy four youth completed an fMRI task involving receipt of social feedback. Region-of-interest analyses examined ventral striatum (VS) activity during positive feedback. Mediation and moderation effects were examined using linear regressions. Sleep disturbance mediated the association between identity-related victimization and SI: higher victimization was associated with worse sleep disturbance, predicting more severe SI. Moderation analyses revealed a positive association between victimization and sleep disturbance at lower but not higher levels of VS response to social reward. Sleep disturbance occurring in the context of social stress heightens vulnerability for SI, particularly among minoritized youth. Greater neural sensitivity to social reward buffers against the effects of victimization on sleep, with implications for mitigating SI. Findings suggest potential mechanisms and individual difference factors underlying minority health disparities.

## Introduction

Suicide is the 2nd leading cause of death among youth in the United States, with rates rising over the past decade (Centers for Disease Control and Prevention, 2023). Youth with minoritized identities, such as those identifying as sexual, gender, and/or racial/ethnic minorities, face heightened risk for suicide (di Giacomo et al., 2018; Marshal et al., 2011; Platt et al., 2022). Almost half of sexual minority youth (SMY) have experienced suicidal ideation (SI), with rates higher among those who also identify as racial/ethnic minorities (“dual minorities”) (The Trevor Project, 2024). Minority stress theory (Meyer, 2003) posits that disparities in SI can be attributed to sexual orientation stigma, including stressors that are external/distal (e.g., discrimination, victimization) and internal/proximal (e.g., internalized shame) (de Lange et al., 2022; Pellicane and Ciesla, 2022). This framework has also been applied towards understanding racial/ethnic minority health disparities (Cyrus, 2017; Wang et al., 2021). Importantly, adolescence is a vulnerable period for suicide due to ongoing development of neural social circuits alongside shifts in sleep and circadian processes, both of which may heighten interpersonal sensitivity and SI risk (Blakemore, 2008; Goldstein and Franzen, 2022; Schriber and Guyer, 2016). These changes are particularly salient during middle-to-late adolescence, given increasing independence and social changes (e.g., transitioning to high school/college/first job). Therefore, it is vital to examine how adolescent sleep patterns and neurobiology may explain the influence of minority stress on SI at this life stage, as both offer opportunities for intervention.

Sleep disturbance is a transdiagnostic process that is impacted by stress and proximally associated with SI (Goldstein and Franzen, 2022; Porras-Segovia et al., 2019). Sleep disturbances are heightened during adolescence and exacerbated among youth with poorer mental health (Gradisar et al., 2011; Hysing et al., 2022). Sleep disturbance refers to disruptions in the 24-h sleep–wake cycle and are typically assessed via self-report measures of sleep quality (Yu et al., 2012). Disturbed sleep is common following acute stress exposure and is a prominent symptom across mental disorders (e.g., mood, anxiety, and trauma and/or stressor-related disorders) (American Psychiatric Association, 2013). Stressful experiences may impact sleep via alterations in cognitive, emotional, and physiological arousal. For instance, exposure to threatening social experiences, such as victimization (e.g., bullying, humiliation) related to sexual orientation, gender, race/ethnicity, and/or body size, can lead to increased fear, worry, and vigilance that interferes with one’s ability to relax and fall/stay asleep (Chan and Fung, 2021; Himmelstein et al., 2019; Lepore and Kliewer, 2013). Because minoritized youth are more likely to experience victimization due to their marginalized identity, they may be especially vulnerable to developing sleep disturbances that may, in turn, enhance risk for mental health problems (de Lange et al., 2022; Jackman et al., 2020; Patterson and Potter, 2019; Sapouna et al., 2023).

Developmental shifts in sleep and circadian processes during adolescence make sleep disturbance a particularly promising candidate for understanding suicide risk in youth. Sleep disturbances stemming from a combination of biological (e.g., decreased sleep drive, delayed melatonin onset) and social changes (e.g., school start times, adjustment to college and/or work schedules) may confer background vulnerability (Goldstein and Franzen, 2022). Along with heightened interpersonal sensitivity during this life stage, exposure to social threats can exert physiological stress that aggravates sleep disturbance and SI. Specifically, experiences of social stress, such as victimization, are cross-sectionally and prospectively associated with self-reported sleep disturbances in youth (Tu et al., 2019; Van Geel et al., 2016). Sleep disturbances, in turn, have been associated with more severe SI (Goldstein et al., 2008; Liu et al., 2020). This could be attributed to deficits in cognitive control and emotion regulation following sleep deprivation and circadian misalignment in sleep–wake activity, which may exacerbate reactivity to social stress and vulnerability for SI (Hamilton et al., 2023; McMakin et al., 2016; Tubbs et al., 2022).

Despite known SI disparities, only recently has research begun to examine associations between social stress, sleep, and SI in minoritized youth. Among Chinese adolescents, sexual minority status was associated with poorer sleep quality, predicting higher odds of SI and suicide attempts (Huang et al., 2018). In the U.K., general victimization experiences were prospectively associated with sleep disruptions that predicted depression and self-injurious behaviors over 6 years in SMY (Tepman and Wong, 2024). Among U.S. racial/ethnic minority youth, shorter sleep duration (<8-h/night) was associated with increased odds of suicidal behaviors (e.g., attempts, suicide plan) (Joseph et al., 2023). These preliminary findings suggest that sleep disturbance from social stress that is related to marginalized identity may contribute to suicide disparities. Given increased neural sensitivity to social feedback during adolescence, it is critical to examine neurobiological characteristics that may influence the effects of social stress on sleep, with potential downstream impact on SI.

Differences in neural reward processing may influence how individuals respond to positive social interactions despite challenging social experiences, with corresponding effects on mental health (Eckstrand et al., 2022a; Forbes et al., 2021). Despite enhanced vulnerability to social stress, adolescence is also a time to develop positive and supportive social experiences (Benner et al., 2017; Roach, 2018). Such meaningful encounters are especially pertinent for minoritized youth (e.g., SMY or racial/ethnic minorities), given their unique experiences of minority stress and the importance of affirming experiences (e.g., identity acceptance or belonging) with family and peers. In turn, these rewarding social interactions may buffer against the impact of minority stress on sleep and suicide risk (Chen et al., 2022; De Lange et al., 2023).

There is robust evidence suggesting that activity in the ventral striatum (VS), which functions as a hub for reward processing and supports goal-directed behaviors––is essential to consider as a characteristic that could confer vulnerability or buffer against stress exposure (Pizzagalli, 2014). Blunted reward-related activity in the VS has been associated with depression and low positive affect or anhedonia, both of which are linked to SI (Avinun et al., 2017; Forbes and Dahl, 2012). Importantly, greater VS response to reward may buffer against the effects of stress on youth mental health and sleep quality (Corral-Frías et al., 2015; Nikolova et al., 2012; Sollenberger et al., 2023). Therefore, regardless of challenging social experiences, youth with enhanced VS response to reward may experience less sleep disturbance and reduced risk for mental health problems. Notably, reward-related research has chiefly examined VS response to *non-social* reward (e.g., money). Given the influence of social factors on minority health disparities, it is crucial to investigate *social* reward processing, which could suggest novel targets for intervention (Eckstrand et al., 2019; Seah et al., 2024).

## Current study

The present research aims to examine the roles of sleep disturbance and social rewarding processing in the relationship between minority stress and SI in a diverse sample of youth at varying suicide risk as indexed by recent and past SI severity, of whom 50% identify as a sexual, racial, and/or Hispanic-ethnicity minority. We conducted a cross-sectional examination of the associations between self-reported identity-related victimization, sleep disturbance, and SI. Unlike prior research, we used a composite measure of identity-related victimization that assessed a range of experiences related to different marginalized identities, including sexual orientation, race or ethnicity, gender, and body size. Based on previous research, we hypothesized that more victimization will be associated with higher SI severity. Additionally, we hypothesized that this association between victimization and SI will be mediated by sleep disturbance: higher victimization will be associated with worse sleep disturbance, predicting more severe SI. Then, we examined if differences in neural responsivity to social reward, as indexed by VS activity during a peer social feedback task, moderated the relationship between victimization and sleep disturbance.

## Methods

### Participants

Participants were recruited as part of a larger study examining the development of affective symptoms during adolescence. Ninety-eight youth (aged 14–22 years; 62% female; 50% sexual minority; 50% racial and/or Hispanic ethnicity minorities) were included here as they had available data for analyses. The larger study comprised two subsamples of participants at varying psychiatric risk: (1) heterosexual youth selected based on presence/absence of familial risk (i.e., first-degree relative) for affective disorders (i.e., mood and schizophrenia-spectrum disorders) and (2) youth selected based on sexual minority identity (i.e., non-heterosexual sexual orientation). In the present study, and consistent with the larger study’s design, 72% of heterosexual youth from the familial-risk subsample had familial history of affective disorders.

The familial-risk and SMY subsamples included in the current study were matched based on self-reported sex assigned at birth and race/ethnicity (Table 1). The total sample comprised 50% (*n* = 49) racial and/or Hispanic-ethnicity minorities, 50% (*n* = 49) sexual minorities, 28% (*n* = 27) heterosexual racial/ethnic minorities, and 24% (*n* = 24) “dual minorities” (sexual minority and racial/ethnic minority). Of these 98, *n* = 74 (62% female; 52% SMY; 46% racial/ethnic minority; 27% dual minorities) had available fMRI data without excessive motion during scanning and at least 50% VS coverage for analysis.

**Table 1:** Sample demographics and primary outcome variables.

Sample characteristic	Total sample (*n* = 98)	SMY (*n* = 49)	Non-SMY (*n* = 49)	Tests of significance
Age (M, SD)	17.20 (2.39)	18.51 (2.38)	15.90 (1.53)	*t*(64.11) = 2.72, *p* < 0.001
Sex assigned at birth (% Female)	62%	69%	55%	*χ^2^* (1, *n* = 98) = 2.13, *p* = 0.145
Sexual orientation				*χ^2^* (4, *n* = 98) = 98.00, *p* < 0.001
100% homosexual (%)	11%	23%	0%	
Mostly homosexual (%)	7%	14%	0%	
Bisexual (%)	21%	43%	0%	
Mostly heterosexual (%)	10%	20%	0%	
100% heterosexual (%)	50%	0%	100%	
Race				*χ^2^* (3, *n* = 98) = 3.77, *p* = 0.287
% Asian	7%	10%	4%	
% Black/African American	32%	31%	33%	
% White	50%	53%	47%	
% Other or multiracial	11%	6%	16%	
Ethnicity (% Hispanic)	7%	6%	7%	*χ^2^* (1, *n* = 88) = 0.03, *p* = 0.862
Current/Past psychiatric disorder (%)^1^	13%	18%	8%	*χ^2^* (1, *n* = 98) = 2.22, *p* = 0.136
Clinical and social characteristics (M, SD, range)^2^				
Overall identity victimization	7.94 (1.93) Range: 6—16	8.43 (2.36) Range: 6—16	7.45 (1.22) Range: 6—11	*F*(1, 95) = 8.53, *p* = 0.004, *η*^2^ = 0.08
Victimization by sexual orientation	8.42 (2.89) Range: 6—21	9.53 (3.61) Range: 6—21	7.31 (1.14) Range: 6—10	*F*(1, 95) = 24.13, *p* < 0.001, *η*^2^ = 0.20
Victimization by race or ethnicity	8.39 (2.35) Range: 6—16	8.55 (2.57) Range: 6—16	8.22 (2.11) Range: 6—14	*F*(1, 95) = 0.38, *p* = 0.541, *η*^2^ = 0.004
Victimization by gender	7.43 (2.46) Range: 6—17	7.90 (2.96) Range: 6—17	6.96 (1.73) Range: 6—13	*F*(1, 95) = 1.36, *p* = 0.247, *η*^2^ = 0.01
Victimization by body size	7.53 (2.90) Range: 6—19	7.76 (3.17) Range: 6—19	7.31 (2.63) Range: 6—16	*F*(1, 95) = 2.73, *p* = 0.102, *η*^2^ = 0.03
Sleep disturbance	50.38 (7.88) Range: 29—71	51.82 (7.89) Range: 29—66	48.94 (7.68) Range: 33—71	*F*(1, 95) = 6.75, *p* = 0.011, *η*^2^ = 0.07
Suicidal ideation^3^	19.68 (23.98) Range: 0—103	25.85 (26.46) Range: 0—103	13.51 (19.61) Range: 0—101	*F*(1, 95) = 12.59, *p* = 0.001, *η*^2^ = 0.12

SMY, Sexual minority youth; Victimization was measured with a 23-item victimization questionnaire (Dermody et al., 2016); Sleep disturbance was measured with the PROMIS Sleep Disturbance-Short Form (Yu et al., 2012); Suicidal ideation was measured with the Suicidal Ideation Questionnaire (Reynolds, 1987).

1 Psychiatric history was determined by whether participants met criteria for a disorder based on the Kiddie Schedule for Affective Disorders and Schizophrenia–Present and Lifetime Version (Kaufman et al., 1997).

2 Tests of significance controlled for age as covariate.

3 Scores reported here are untransformed values for ease of interpretation.

Participants were recruited from the community using flyers, research registries, other research studies, and outpatient clinics, including those with a higher proportion of SMY. Participants and their first-degree relative (parent and/or sibling) in the familial-risk subsample completed diagnostic interviews (Kiddie Schedule for Affective Disorders and Schizophrenia–Present and Lifetime Version; K-SADS-PL; Kaufman et al., 1997) to determine and confirm psychiatric risk classification and eligibility. As part of eligibility criteria for study enrollment, participants from the familial risk subsample who were taking psychotropic medications were excluded. Psychotropic medication use was allowed for the SMY subsample, although details were not assessed, which could limit interpretation of findings. All study procedures were approved by the university IRB, and participants (and their parent/guardian if <18 years old) provided informed consent.

### Procedure

**Figure 1:**
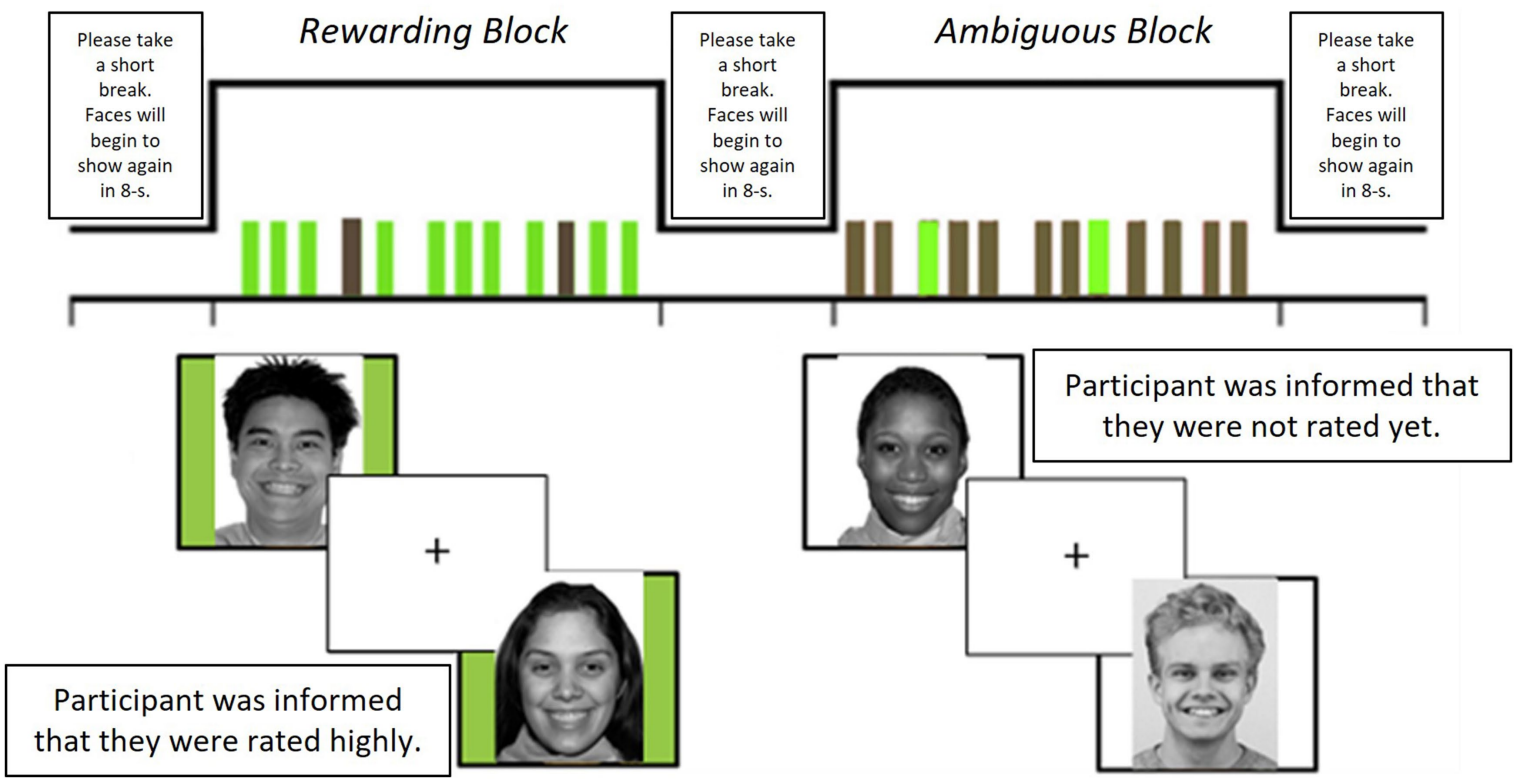
Description of fMRI task design. Photos with green-colored background indicate rewarding social feedback while those with white-colored background indicate ambiguous feedback. Task procedure: Before scanning, participants rated 40 photos (50% female) of other youth (age-matched to participants) based on how much they would like these peers (rated 1-“not at all” to 9-“very much”). Participants were told that these peers were other participants in the research study and were unaware that these photos were from a standardized database of facial stimuli. Participants had their photos taken, as part of a cover story, and were informed that they would also be rated by other unfamiliar “peers.” Each participant had a personalized stimulus set of 32 photos based on their likeability ratings: 16 photos with rewarding feedback (i.e., informed that they were rated positively by other peers) and 16 with ambiguous feedback (i.e., informed that peers had not rated them yet). To increase salience of social feedback, rewarding feedback comprised the eight highest-rated (mutual liking) and eight lowest-rated (received liking) photos (50% female) and ambiguous feedback comprised 16 moderately-rated photos (50% female) for each participant. Task design: A block design comprising four rewarding and four ambiguous feedback blocks was used to present stimuli. Each photo appeared three times over the eight blocks. Each photo provided either rewarding or ambiguous feedback but not both, and order of stimulus presentation was counterbalanced. Each block comprised 12 photos, lasting for 84-s. To reduce habituation and predictability of the block design, 2 of the 12 photos in each block were of the opposite stimulus type (e.g., 2 rewarding feedback stimuli in the ambiguous block). Each photo was presented for 3-s with a jittered inter-stimulus interval (1, 3, 5, or 7 s), and blocks were separated by an 8-s interval. Participants were instructed to press a button whenever they saw each photo as an attention check during the task. After scanning, participants were debriefed on task deception and informed that the facial stimuli were derived from a standardized database (Martinez and Benavente, 1998) and that their photos were not actually rated by peers.

Baseline assessment data for the familial-risk and SMY^1^ subsamples were included in analyses. Participants completed self-report measures of demographics, exposure to identity-related victimization events, sleep disturbance, and SI (means, SDs, and range in Table 1). Following, participants attended an fMRI session and completed a social reward task (Figure 1).

### Measures

#### Sexual minority status

Sexual minority status was determined by a single-item measure as in the National Longitudinal Study of Adolescent Health: “100% Heterosexual (Straight),” “Mostly Heterosexual (Straight but somewhat attracted to people of the same sex),” “Bisexual (Attracted to men and women equally),” “Mostly Homosexual (Gay, but somewhat attracted to the opposite sex),” “100% Homosexual (Gay or Lesbian),” “Do not Know,” or “Not Listed.” Participants endorsing any option besides “100% Heterosexual (Straight)” were classified as SMY. No participant endorsed “Do not Know” or “Not Listed.”

#### Racial/ethnic minority status

Race was assessed with seven possible options: (1) Black or African American, (2) Asian, (3) White, (4) Native Hawaiian or Pacific Islander, (5) Native American or Alaskan Native, (6) More than One Race, (7) or Other. Participants also indicated whether they were of Hispanic ethnicity. In our analyses, participants who indicated a race other than “White” or Hispanic ethnicity were classified as racial/ethnic minorities.

#### Sex assigned at birth

A free-text response option was provided for participants to indicate their sex. All participants indicated either “female” or “male.”

#### Exposure to identity-related victimization events

A 23-item questionnaire (Dermody et al., 2016) assessed exposure to identity-related victimization events (1. bullied, 2. hit/beaten up, 3. treated unfairly, 4. called hurtful or insulting names, 5. witnessing others being hit/beaten up, and/or 6. witnessing others being called hurtful or insulting names) based on sexual orientation, race or ethnicity, gender, and/or body size in the past 6 months. Each item, participants responded on a 4-point scale (“1-Never” to “4-Many Times”). Average frequency of exposure to recent identity-related victimization was based on the mean score across identity categories (Cronbach’s *α* = 0.86). Subscale scores are also in Table 1.

#### Sleep disturbance

The 8-item Patient Reported Outcomes Measurement Information System (PROMIS^®^) Sleep Disturbance—Short Form 8a (Yu et al., 2012) assessed past-week sleep disturbances. Items assessed overall sleep quality and satisfaction, difficulties falling and staying asleep, and restless sleep. Item scores were summed and converted to T-scores (mean = 50, SD = 10) (Cronbach’s α = 0.89).

#### Suicidal ideation

The 30-item Suicidal Ideation Questionnaire (SIQ) (Reynolds, 1987) assessed past-month SI severity, ranging from passive thoughts about death to serious thoughts of actively engaging in suicidal behaviors. An index of SI was obtained by calculating the sum across items (Cronbach’s α = 0.96). In the present study, 14% of the sample scored ≥41, which is the cut-off score indicating high clinical risk. Additionally, 54% of the sample endorsed at least one of eight critical SIQ items examining suicidal intent, method, and plan.

To characterize past-year SI severity, we examined participants’ responses to four items assessing past-year suicidal behaviors in the 2015 Youth Risk Behavior Survey (see Supplementary material). Nine indicated seriously considering suicide, four made a suicide plan and/or attempt, and one suffered severe injuries because of their suicide attempt.

#### fMRI social reward task

Neural reactivity to social reward was examined using a block-design fMRI task (Healey et al., 2014). Task procedures are detailed in Figure 1. Across eight blocks, participants viewed photos of other age-matched unfamiliar youth (50% female) of varying race/ethnicity whom participants had previously rated regarding likeability before scanning. While viewing these photos, participants were informed if they were rated positively by these youth (rewarding condition; four blocks) or not rated yet, which is open to interpretation (ambiguous condition; four blocks). In the current study, we focused our analyses on the rewarding condition.

### fMRI acquisition parameters and preprocessing

Scanning was performed in a Siemens 3 T trio scanner and fMRI acquisition parameters followed current convention: MPRAGE structural images with high-resolution T1-weighted images with 1 mm isometric voxels (TR/TE/flip angle = 1,500 ms/3.19 ms/8°; FOV = 256 × 256; 176 continuous slices) and field maps (2.3 mm isotropic voxels; TR/TE1/TE2/flip angle = 550 ms/4.92 ms/7.38 ms/50°; FOV = 220 × 220; bandwidth 380 Hz/Px) were obtained. Functional blood-oxygen-level-dependent (BOLD) images were acquired using multi-band gradient echo planar imaging (EPI) sequences: 18 slices, three-factor multi-band; 2.3 mm isotropic voxels; TR/TE/flip angle = 1,500/30 ms/58°, FOV = 220 × 220, matrix = 96 × 96; bandwidth 1736 Hz/Px. A reference EPI scan obtained before fMRI data collection was visually inspected for artifacts and signal quality.

SPM12 was used to perform preprocessing and fMRI image analysis. BOLD images for each subject were realigned to the mean volume in the time series and co-registered with the subject’s structural image. Image distortion was corrected using field maps. Structural images were normalized via a non-linear transformation to the standard MNI/ICBM 152 tissue probability maps and segmented into gray and white matter, cerebrospinal fluid, and other tissues. BOLD images were transformed into the same space using the structural image and resampled at 2 mm^3^ isotropic voxel size. BOLD images were normalized and spatially smoothed (FWHM 6 mm).

### Data analytic strategy

#### Neuroimaging analyses

First-level analyses examined participants’ neural reactivity to socially rewarding feedback. A fixed-effect general linear model (GLM) was performed for each participant, which included the rewarding blocks, ambiguous blocks, and baseline fixation. Neural response to rewarding feedback was determined for Rewarding>Baseline. Volumes with high motion and artifacts were identified using ART (average image intensity deviated >3SD from the mean intensity or where movement exceeded 0.5 mm in translation or 0.01° in rotation from the previous image) (Chai et al., 2014), which was used to create an additional regressor in the first-level GLM to reduce motion-related noise. The six realignment parameters determined during preprocessing were entered as covariates to control for head movement. A 128 s high-pass filter and autoregressive modeling were implemented during fitting.

One-sample *t*-tests examined bilateral VS activity during social reward (vs. baseline). The VS mask was derived from the Neurosynth meta-analytic database (using the association test map at 50% threshold to constrain the mask to VS and exclude surrounding regions). VS activation to socially rewarding feedback was determined by extracting the principal eigenvariate in the left/right VS per participant. Principal eigenvariate values were winsorized to 3SDs of the sample mean to reduce influence of outliers (1.4% [*n* = 1] of participant values). These values were then averaged to index mean VS response to social reward and used in statistical analyses.

### Statistical analyses

To test for statistical mediation of the relationship between victimization and SI by sleep disturbance, a series of linear regression analyses of the total effect (*c*), direct effect (*c*’), and bootstrapped bias-corrected 95% confidence intervals of the indirect effect (*ab*) were computed for each predictor variable using SPSS Hayes PROCESS (Model 4) with 5,000 bootstrapped samples (Preacher and Hayes, 2008). Mean victimization (related to sexual orientation, gender, race or ethnicity, and body size) was entered as the predictor, SI as the outcome, and sleep disturbance as the mediator. Significant mediation is observed if the confidence intervals of the indirect effect do not contain zero.

Moderation analyses utilized Hayes PROCESS (Model 1). For testing two-way moderation, victimization was entered as the predictor, mean VS activation to social reward was entered as the moderator, and sleep disturbance was entered as the outcome. The Johnson-Neyman technique was used to probe significant moderation effects by determining the region(s) of significance, i.e., value(s) of VS activity where significant associations existed between victimization and sleep disturbance (Bauer and Curran, 2005). Sexual (1 = SMY, 0 = non-SMY) and racial/ethnic (1 = racial/ethnic minority, 0 = White) minority status and self-reported sex (1 = Female, 0 = Male,) were included as covariates in both mediation and moderation analyses. All predictor variables were mean centered.

## Results

### Preliminary analyses

The SMY and familial-risk subsamples did not differ in self-reported sex, race/ethnicity, and lifetime psychiatric disorder diagnosis based on the K-SADS-PL, although SMY were older (Table 1). For the whole sample, SIQ scores were positively skewed (skewness = 1.79) and subsequently square-root transformed to normalize the data distribution (skewness = 0.58). Bivariate correlations indicated that higher SI was associated with greater sleep disturbance (*r* = 0.37, *p* < 0.001) and more victimization (*r* = 0.37, *p* < 0.001). More victimization was associated with greater sleep disturbance (*r* = 0.31, *p* = 0.002). Age was not associated with victimization (*r* = 0.03, *p* = 0.769), sleep disturbance (*r* = −0.06, *p* = 0.577), and SI (*r* = −0.02, *p* = 0.813).

### Group differences in clinical outcomes

One-way ANCOVA (controlling for age) indicated that the SMY subsample had more severe victimization (*p* = 0.004), sleep disturbance (*p* = 0.011), and SI (*p* = 0.001) (Table 1). Notably, SMY (22%) were more likely than heterosexual youth (6%) to score above the SIQ clinical cut-off score (≥41), *χ*^2^ (df = 1, *n* = 98) = 5.33, *p* = 0.021, and endorse more SIQ critical items (SMY: M = 3.16, SD = 2.89, heterosexual: M = 1.09, SD = 1.77), *F*(1, 95) = 18.43, *p* < 0.001.

Additional analyses examining group differences based on race/ethnicity and sex are reported in Supplementary Table S1. Racial/ethnic minorities reported lower SI severity than White peers (*p* = 0.023). Female and male youth reported similar SI severity (*p* = 0.119). There were no group differences in self-reported mean victimization or sleep disturbance by race/ethnicity (*p* = 0.515; *p* = 0.903) or sex (*p* = 0.061; *p* = 0.334), although racial/ethnic minorities and female youth reported greater victimization due to race/ethnicity and gender, respectively (Supplementary Table S1).

### Mediation analysis^2^

**Figure 2:**
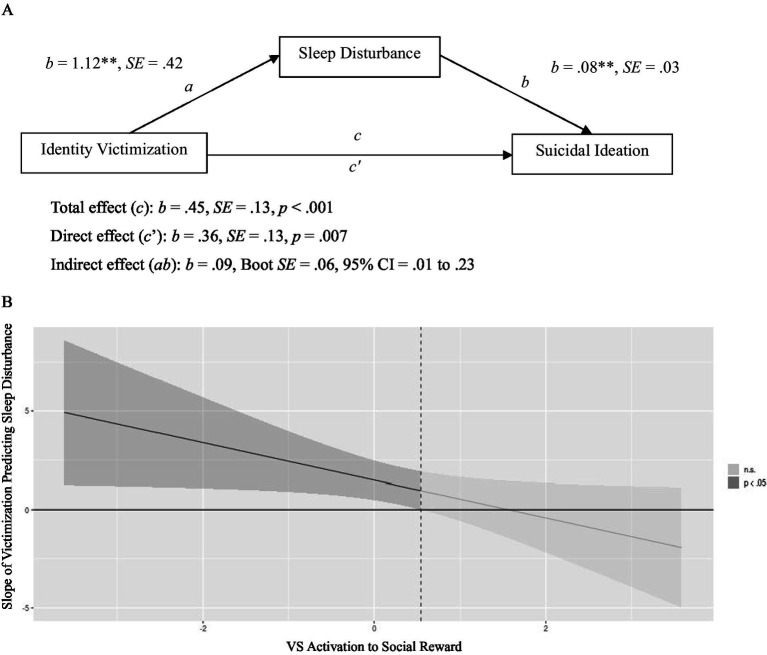
(A) Describes the mediation of identity victimization and suicidal ideation by sleep disturbance. All regression coefficients presented are unstandardized. (B) The Johnson-Neyman regions of significance plot depicting the interaction between identity victimization and ventral striatum (VS) activation to social reward in predicting sleep disturbance. The line represents slope coefficients (between identity victimization and sleep disturbance) across values of the moderator (VS activation). (A) Mediation of Identity Victimization and Suicidal Ideation by Sleep Disturbance. **p < 0.01; CI = confidence interval. (B) Identity Victimization × VS Activation to Social Reward Predicting Sleep Disturbance. Darker shaded regions indicate region(s) of significance using the Johnson-Neyman technique, i.e., range of VS activation values (x-axis) when the slope between identity victimization and sleep disturbance (y-axis) is significantly different from zero (*p* < 0.05). n.s. = not significant (*p* > 0.05).

Sleep disturbance mediated the relationship between victimization and SI, indirect effect: 0.09, BootSE = 0.06, 95% CI [0.01, 0.23], total effect: 0.45, SE = 0.13, *p* < 0.001 direct effect: 0.36, SE = 0.13, *p* = 0.007 (Figure 2A). Specifically, more victimization was associated with greater sleep disturbances (*p* = 0.009), which were, in turn, associated with more severe SI (*p* = 0.009).

### Moderation analysis^3^

VS activation to social reward moderated the relationship between victimization and sleep disturbance, B = −0.95, *p* = 0.038 (Table 2). The Johnson-Neyman technique indicated that victimization was positively associated with sleep disturbance at lower but not higher levels of VS activity to rewarding social feedback (principal eigenvariate<0.55, *p* < 0.05; 73% of the sample; shaded in Figure 2B).

**Table 2:** Two-way (victimization × VS activity to social reward) interaction in predicting sleep disturbance (*n* = 74).

Predictor	*B*	*SE*	*t*	*p*	95% CI	*R*^2^	Test of Significance
Sexual minority status	1.45	1.78	0.82	0.418	−2.10 to 5.01	0.20	*F*(6, 67) = 2.72, *p* = 0.020
Sex assigned at birth	0.22	1.79	0.12	0.901	−3.36 to 3.80		
Racial/Ethnic minority status	−1.69	1.77	−0.96	0.341	−5.22 to 1.83		
Identity victimization	1.49	0.50	2.98	0.004	0.49 to 2.49		
VS activity	0.84	0.84	1.00	0.321	−0.84 to 2.52		
Identity victimization × VS activity	−0.95	0.45	−2.12	0.038	−1.85 to −0.05		

VS, ventral striatum; Victimization was measured using the 23-item Victimization Questionnaire (Dermody et al., 2016).

## Discussion

The present study investigated sleep disturbance and social reward processing as characteristics underlying the relationship between identity-related victimization and SI in a diverse adolescent sample. Consistent with prior data, we found that SMY (vs. heterosexual youth) reported more frequent victimization experiences, worse sleep, and more severe SI. Group differences in SI and sleep disturbance based on race/ethnicity or sex were inconsistent, suggesting that disparities in these mental health outcomes are more pronounced for SMY. Notably, we found that sleep disturbance mediated the association between victimization and SI, where greater exposure to identity-related victimization events was associated with increased sleep disturbance that, in turn, predicted worse SI. Moderation analyses indicated that victimization was associated with poorer sleep but only at lower VS response to social reward. These findings suggest that VS activation to social reward buffers against the effects of victimization on sleep, with potential implications for mitigating SI.

In line with previous research, we found that SMY reported more frequent exposure to victimization experiences (Priebe and Svedin, 2012; Tepman and Wong, 2024). Critically, unlike prior work, we assessed victimization experiences related to several pertinent aspects of identity. Therefore, we were able to examine how various identity-specific social stressors influenced mental health: More frequent experiences of identity victimization were associated with worse mental health outcomes, including poorer sleep quality and more severe SI. These findings align with developmental theories regarding adolescents’ susceptibility to social contexts and corresponding risk for psychopathology (Forbes et al., 2021; Schriber and Guyer, 2016), as well as minority stress theory, where experiences of distal/external minority stressors, such as victimization, contribute to the development of adverse health outcomes (Eckstrand et al., 2022a; Meyer, 2003).

Supporting our hypothesis, sleep disturbance mediated the relationship between victimization and SI (Huang et al., 2018; Tepman and Wong, 2024). Because victimization experiences were self-reported as occurring in the prior 6 months, while sleep disturbances were about the preceding week, our findings suggest that sleep disruptions may accumulate following stressful social experiences and exacerbate SI. However, more frequent sampling in longitudinal studies would be necessary to confirm prospective relationships. Increased vigilance or worry about physical or psychological safety following victimization may contribute to difficulties falling/staying asleep and poor sleep quality. In turn, sleep deprivation may disrupt cognitive control and emotion regulation ability and exacerbate SI (Ben Simon et al., 2020; Porras-Segovia et al., 2019; Tubbs et al., 2022). Indeed, recent research suggests that sleep disruptions worsen next-day SI via altered emotional reactivity to social interactions (Hamilton et al., 2023). Taken together, these findings suggest the possibility of a negative spiral where experiences of social stress contribute to sleep disturbances, which may inhibit effective emotion regulation and aggravate SI. Future studies could consider testing this hypothesis using serial mediation with longitudinal data.

Separately, sleep disturbance may also be a symptom indicative of other mental health problems, such as mood, anxiety, or stressor-related disorders, that explain heightened risk for suicide (Harvey, 2008). Although we hypothesized that sleep disturbance precedes SI, the alternative may also be true: SI stemming from victimization could lead to sleep problems, suggesting a possible reciprocal relationship. Indeed, distress and rumination related to SI could lead to greater sleep disturbances, including poorer sleep quality, longer sleep onset latency, and shorter sleep duration (Clancy et al., 2020). Due to the cross-sectional nature of our data, however, we were unable to test the directionality of these associations, but this would be an important future direction. Clinically, as assessment of self-reported sleep disturbance is convenient and less stigmatizing, our findings suggest that incorporating these assessments in routine care or schools could be helpful in risk identification, particularly among victimized youth. In addition, sleep is a modifiable risk factor (Harvey, 2008); our findings indicate that sleep interventions could be beneficial for mitigating the occurrence and/or severity of SI (Hamilton and Buysse, 2019).

Results from moderation analyses revealed that individual differences in social reward processing, as indexed by VS activation to rewarding social feedback, buffers against the effects of victimization on sleep. These findings extend prior research by our team. Using data from the same study, Eckstrand et al. (2022b) found that sexual orientation victimization was associated with more severe depression symptoms, but only among youth with dampened response to monetary reward. Seah et al. (2024) found that dampened activity in the temporoparietal junction—a key brain region for social cognition and reward processing (Saxe and Kanwisher, 2003)—to social reward was associated with worse SI in SMY vs. heterosexual youth. Altogether, these findings indicate that diminished neural reward response may underlie disparities across a broad range of youth mental health outcomes. Notably, research examining reward processing and mental health in racial/ethnic minority youth is lacking, highlighting this as a key area for further study. Our findings suggest that enhancing individuals’ sensitivity and responsivity to reward (e.g., savoring) may benefit sleep health despite challenging social experiences. This could be particularly important for minoritized youth who tend to experience considerable social disadvantage. Increasing evidence suggests that enhancing *positive* affect via psychosocial interventions can lead to beneficial outcomes distinct from traditional treatments focused on decreasing *negative* affect (Coifman et al., 2021; Craske et al., 2023). Future research should examine how these interventions may influence and/or interact with neural reward circuitry to impact mental health.

Strengths of the current study include a risk-enriched sample of youth with diverse sexual orientation and racial/ethnic identities, the use of well-validated questionnaires, and neuroimaging methodology. The focus on sleep and neurobiology in the relationship between minority stress and suicide is also a novel and understudied topic. Nonetheless, our findings should be considered with the following limitations. First, the cross-sectional study design prevents conclusive interpretations surrounding directionality of effects. While our moderated mediation findings represent one interpretation of the results, it may also be the case that blunted responses to social reward is caused by a combination of heightened sleep disturbance and SI (McMakin et al., 2016; Pechtel et al., 2013). Therefore, future research using longitudinal data is needed to examine prospective associations of social experiences, sleep disturbance, and SI. Second, we relied solely on a self-report measure of sleep disturbance. Although subjective measures can provide meaningful information regarding sleep quality, future research should consider incorporating objective measures such as daily actigraphy and/or polysomnography (e.g., ZMax headband; Buysse, 2014; Esfahani et al., 2023). Doing so can allow examination of other sleep features uniquely associated with suicide risk. Third, the frequency of reported victimization and severity of SI in our sample was relatively limited, as the questionnaires only assessed recent experiences. Future studies should consider collecting more comprehensive data, such as lifetime history of victimization and/or suicidal behaviors. Finally, although we used a risk-enriched sample, it would be important to examine if our findings replicate in samples with more severe suicidal ideation and behavior.

Overall, our findings have potential to improve understanding of biological pathways underlying minority stress and suicide, with clinical implications for alleviating health disparities. We found that identity-related victimization was associated with more severe SI through its detrimental effects on subjective experiences of sleep disturbance. Importantly, greater VS activation to social reward mitigated the impact of victimization on sleep health. Our findings suggest that sleep and social reward processing may be relevant features for suicide detection and prevention, particularly among victimized youth.

## Data Availability

The raw data supporting the conclusions of this article will be made available by the authors, without undue reservation.

## Footnotes

^1^The SMY subsample only provided one timepoint of data.

^2^Alternatively, we examined sexual minority status as a predictor in the mediation model (in place of victimization). Sleep also emerged as a significant mediator in these analyses (Supplementary Figure S1).

^3^Additional analyses examining VS activation as a moderator of the association between sexual minority status and sleep disturbance were not significant (Supplementary Table S2).
