# Supplementary material for: Sleep disturbance and social reward processing as characteristics linking minority victimization and suicidal ideation in youth
Source: Front Neurosci. 2025 Jan 7;18:1475097. doi: 10.3389/fnins.2024.1475097 (PMC11747665; doi:10.3389/fnins.2024.1475097)
Supplement: Supplementary file 1 [file Data_Sheet_1.docx]

**Sleep Disturbance and Social Reward Processing as Characteristics Linking Minority Victimization and Suicidal Ideation in Youth**

T. H. Stanley Seah^1^, Kristen L. Eckstrand^1^, Tina Gupta^1^, Lily C. X. Jensen^2^, Zachary M. Brodnick^1^, Chloe M. Horter^1^, Alice M. Gregory^3^, Peter L. Franzen^1^, Michael P. Marshal^1^, & Erika E. Forbes^1^

^1^University of Pittsburgh

^2^Harvard University

^3^Royal Holloway, University of London

**Supplemental Materials**

**Youth Risk Behavior Survey (YRBS) Suicide-Related Items**

| **Suicidal Behavior Items** | |
| --- | --- |
| Q27. During the past 12 months, did you ever seriously consider attempting suicide? | A. Yes  B. No |
| Q28. During the past 12 months, did you make a plan about how you would attempt suicide? | A. Yes  B. No |
| Q29. During the past 12 months, how many times did you actually attempt suicide? | A. 0 times  B. 1 time  C. 2 or 3 times  D. 4 or 5 times  E. 6 or more times |
| Q30. If you attempted suicide during the past 12 months, did any attempt result in an injury, poisoning, or overdose that had to be treated by a doctor or nurse? | A. I did not attempt suicide during the past 12 months  B. Yes  C. No |

**fMRI Acquisition Parameters and Preprocessing**

Participants underwent scanning in a Siemens 3T Trio scanner. MPRAGE structural images with high-resolution T1-weighted images with 1mm isometric voxels (TR/TE/flip angle=1,500ms/3.19ms/8°; FOV=256×256; 176 continuous slices) and field maps (2.3mm isotropic voxels; TR/TE1/TE2/flip angle=550ms/4.92ms/7.38ms/50°; FOV=220×220; bandwidth 380Hz/Px) were obtained. Functional blood-oxygen-level-dependent (BOLD) images were acquired using multi-band gradient echo planar imaging (EPI) sequences: 18 slices, three-factor multi-band; 2.3mm isotropic voxels; TR/TE/flip angle=1,500/30ms/58°, FOV=220×220, matrix=96×96; bandwidth 1736Hz/Px. A reference EPI scan obtained before fMRI data collection was visually inspected for artifacts and signal quality.

SPM12 (<http://www.fil.ion.ucl.ac.uk/spm>) was used to perform preprocessing and fMRI image analysis. BOLD images for each subject were realigned to the mean volume in the time series and co-registered with the subject’s structural image. Image distortion was corrected using field maps. Structural images were normalized via a non-linear transformation to the standard MNI/ICBM 152 tissue probability maps and segmented into gray and white matter, cerebrospinal fluid, and other tissues. BOLD images were transformed into the same space using the structural image and resampled at 2mm^3^ isotropic voxel size. BOLD images were normalized and spatially smoothed (FWHM 6mm).

***Figure S1.*** Figure S1 describes the mediation of sexual minority status and suicidal ideation by sleep disturbance. All regression coefficients presented are unstandardized.

S1. Mediation of Sexual Minority Status and Suicidal Ideation by Sleep Disturbance

Sleep Disturbance

*a*

*b*

*b* = .10**, *SE* = .03

*b* = 4.86*, *SE* = 2.00

Sexual Minority Status

*c*

Suicidal Ideation

*c'*

Total effect (*c*): *b* = 1.75, *SE* = .63, *p* = .007

Direct effect (*c*’): *b* = 1.26, *SE* = .62, *p* = .046

Indirect effect (*ab*): *b* = .49, Boot *SE* = .25, 95% CI = .07 to 1.05

*Note*. **p* < .05; ***p* < .01; CI = confidence interval

**Table S1**

*Sample Demographics and Primary Outcome Variables*

|  | Racial/Ethnic Minority Youth | White Youth | Tests of Significance | Female Youth | Male Youth | Tests of Significance |
| --- | --- | --- | --- | --- | --- | --- |
| *n* | 50 | 48 |  | 61 | 37 |  |
| Sexual Orientation |  | | *χ^2^* (4, *n* = 98) = 2.68,  *p* = .613 |  | | *χ^2^* (4, *n* = 98) = 14.42,  *p* = .006 |
| 100% Homosexual (%) | 12% | 10% |  | 5% | 22% |  |
| Mostly Homosexual (%) | 4% | 10% |  | 8% | 5% |  |
| Bisexual (%) | 18% | 25% |  | 31% | 5% |  |
| Mostly Heterosexual (%) | 12% | 8% |  | 12% | 8% |  |
| 100% Heterosexual (%) | 54% | 46% |  | 44% | 60% |  |
| Clinical Characteristics (M, SD, Range)^1^ | | | | | | |
| Overall Identity Victimization | 8.08 (2.05)  Range: 6—16 | 7.80 (1.81)  Range: 6—15 | *F*(1, 95) = 0.43,  *p* = .515, *η*^2^ = .004 | 8.22 (2.15)  Range: 6—16 | 7.49 (1.40)  Range: 6—11 | *F*(1, 95) = 3.59,  *p* = .061, *η*^2^ = .04 |
| Sexual Orientation | 7.98 (2.45)  Range: 6—18 | 8.88 (3.25)  Range: 6—21 | *F*(1, 95) = 2.54,  *p* = .114, *η*^2^ = .03 | 8.62 (3.09)  Range: 6—21 | 8.08 (2.53)  Range: 6—16 | *F*(1, 95) = 0.87,  *p* = .353, *η*^2^ = .01 |
| Race or Ethnicity | 8.92 (2.80)  Range: 6—16 | 7.83 (1.62)  Range: 6—12 | *F*(1, 95) = 5.33,  ***p* = .023**, *η*^2^ = .05 | 8.56 (2.51)  Range: 6—16 | 8.11 (2.07)  Range: 6—14 | *F*(1, 95) = 0.94,  *p* = .335, *η*^2^ = .01 |
| Gender | 7.64 (2.62)  Range: 6—17 | 7.21 (2.29)  Range: 6—16 | *F*(1, 95) = 0.35,  *p* = .554, *η*^2^ = .004 | 7.85 (2.85)  Range: 6—17 | 6.73 (1.41)  Range: 6—13 | *F*(1, 95) = 6.55,  ***p* = .012**, *η*^2^ = .07 |
| Body Size | 7.76 (2.97)  Range: 5—18 | 7.29 (2.85)  Range: 5—19 | *F*(1, 95) = 1.00,  *p* = .320, *η*^2^ = .01 | 7.84 (3.00)  Range: 5—19 | 7.03 (2.71)  Range: 5—16 | *F*(1, 95) = 1.43,  *p* = .236, *η*^2^ = .02 |
| Sleep Disturbance | 50.21 (7.81)  Range: 29—71 | 50.56 (8.03)  Range: 33—66 | *F*(1, 95) = 0.15,  *p* = .903, *η*^2^ = .00 | 51.03 (7.44)  Range: 36—66 | 49.31 (8.55)  Range: 29—71 | *F*(1, 95) = 0.94,  *p* = .334, *η*^2^ = .01 |
| Suicidal Ideation^2^ | 13.38 (15.68)  Range: 0—79 | 26.24 (29.06)  Range: 0—103 | *F*(1, 95) = 5.32,  ***p* = .023**, *η*^2^ = .05 | 23.12 (26.41)  Range: 0—103 | 14.01 (18.28)  Range: 0—101 | *F*(1, 95) = 2.47,  *p* = .119, *η*^2^ = .03 |
| Note. SMY = Sexual minority youth; Victimization was measured with a 23-item victimization questionnaire (Dermody et al., 2016); Sleep disturbance was measured with the PROMIS Sleep Disturbance-Short Form (Yu et al., 2011); Suicidal ideation was measured with the Suicidal Ideation Questionnaire (Reynolds, 1987); ^1^Tests of significance controlled for age as covariate; ^2^Scores reported here are untransformed values for ease of interpretation. | | | | | | |

| **Table S2** |  |  |  |  |  |  |  |
| --- | --- | --- | --- | --- | --- | --- | --- |
| Two-way (Sexual Minority Status × VS Activity to Social Reward) Interaction in Predicting Sleep Disturbance (n=74) | | | | | | | |
| Predictor | *B* | *SE* | *t* | *p* | 95% CI | *R*^2^ | Test of Significance |
| Age | -1.38 | .54 | -2.56 | .013 | -2.45 to -.30 | .15 | *F*(6, 67) = 2.02, *p* = .075 |
| Sex Assigned at Birth | -.72 | 1.98 | -0.36 | .718 | -4.67 to 3.23 |  |  |
| Racial/Ethnic Minority Status | .84 | 1.94 | 0.43 | .667 | -3.03 to 4.70 |  |  |
| Sexual Minority Status | 6.00 | 2.20 | 2.73 | .008 | 1.61 to 10.40 |  |  |
| VS Activity | 1.22 | 1.61 | 0.76 | .449 | -1.98 to 4.43 |  |  |
| Sexual Minority Status × VS Activity | .38 | 1.94 | 0.20 | .845 | -3.50 to 4.26 |  |  |
| Note. VS = Ventral striatum. | | | | | | | |
